# Automated Craniofacial Artery Segmentation with Vessel Enhancement-Guided Deep Learning

**DOI:** 10.3390/bioengineering13070728

**Published:** 2026-06-24

**Authors:** Hyeonju Park, Young Chul Kim, Kyoyeong Koo, Sangyun Kang, Jong Woo Choi, Chan-Ung Park

**Affiliations:** 1 SKIA Inc., 1502, 288 Digital-ro, Guro-gu, Seoul 08390, Republic of Korea; hjpark@skia.kr (H.P.);; 2 Department of Plastic and Reconstructive Surgery, Asan Medical Center, Ulsan University College of Medicine, 88 Olympic-ro 43-gil, Songpa-gu, Seoul 05505, Republic of Korea

**Keywords:** arteries, image processing, deep learning, computed tomography angiography

## Abstract

Computed tomography angiography (CTA)-based segmentation of the superficial temporal arteries (STAs) and facial vessels (FVs) is important for neurosurgical and reconstructive planning. Nevertheless, segmentation of STAs and FVs remains challenging because of their small caliber, tortuous courses, and proximity to high-intensity bony structures. This study aims to develop a deep learning framework for accurate automated segmentation of these craniofacial vessels. A single-input 3D nnU-Net v2 model was trained using raw CTA volumes, while a Fusion-based Vesselness Map (FVM) was constructed from multiscale vessel-enhancement filters to emphasize small vascular structures and suppress irrelevant regions such as the skull and skin. Instead of being used as an additional input channel, the FVM was incorporated into the loss function as a spatial prior to guide the network toward vessel boundaries and distal branches. In 72 clinical cases, the FVM-guided model improved segmentation accuracy compared with a baseline model trained with Dice Focal Loss, particularly in boundary delineation. For the STAs, the Average Symmetric Surface Distance decreased from 6.543 mm to 2.941 mm. Qualitative evaluation further showed reduced segmentation noise and fewer false positives near bone and distal branches. These findings suggest that integrating classical vessel enhancement into deep learning supervision can improve morphologically consistent craniofacial vessel segmentation and support preoperative surgical planning.

## 1. Introduction

The superficial temporal arteries (STAs) and facial vessels (FVs) are critical anatomical structures in neurosurgical, maxillofacial, aesthetic and reconstructive surgeries [1,2]. The STAs serve as the primary donor vessels for extracranial-intracranial bypass procedures in Moyamoya disease and complex cerebrovascular pathologies, and the FVs are essential for facial reconstructive procedures and surgical landmark identification [3,4]. Accurate identification of these vessels is essential to prevent severe bleeding, flap necrosis, or ischemic complications during surgical procedures. Although preoperative computed tomography angiography (CTA) is routinely performed, conventional visualization techniques, such as standard volume rendering, often fail to isolate these highly tortuous and thin superficial vessels from adjacent high-density skeletal structures. Moreover, manual segmentation is excessively time-consuming and labor-intensive, making it impractical for routine clinical workflows.

Over the past decade, techniques for the automatic 3D segmentation of arterial structures in CTA images have advanced remarkably [5,6,7]. Early studies in the 2010s established foundational methodologies for coronary and carotid artery segmentation by combining traditional computer vision techniques with machine learning [8,9]. Subsequently, the emergence of Convolutional Neural Networks (CNNs) revolutionized this field, enabling the successful segmentation of comprehensive arterial networks—ranging from the aorta to cerebral vessels—using 3D deep learning architectures [10,11,12,13]. Recently, nnU-Net v2 [14] has emerged as a state-of-the-art framework in medical image segmentation. Unlike traditional methods that require extensive manual tuning of hyperparameters and network architectures, nnU-Net v2 automatically configures the entire pipeline—including preprocessing, network topology, and training schemes—based on the specific properties of the dataset. This self-configuring nature has allowed it to outshine many specialized, manually tuned models in various biomedical challenges.

Recently, research on vascular segmentation has focused on integrating various filtering techniques with machine learning. Survarachakan et al. [15] analyzed the effects of various vesselness enhancement filters and gamma correction preprocessing to improve liver vessel segmentation performance. Their results demonstrated that fusing multiple filters or incorporating gamma correction significantly improved segmentation performance (Dice score: 0.830) compared to applying a single filter. This case suggests that filter fusion techniques optimized for the specific characteristics of target vessels are essential for ensuring the robustness of segmentation algorithms. Huang et al. [16] proposed a methodology combining a Symmetric Reverse Filter (SRF) with D-means clustering for the automatic segmentation of coronary arteries in CTA images. By effectively capturing the gradient change characteristics of vessel boundaries through SRF applied to three orthogonal planes, the study reduced computational costs while achieving high accuracy (Dice Similarity Coefficient, DSC: 0.9318). This suggests that filter designs reflecting geometric properties can contribute to enhancing the discriminative power for vascular tissues. Furthermore, Chen et al. [17] proposed a 3D multi-channel U-Net structure that utilizes both original CTA images and Frangi-based vesselness maps as simultaneous inputs. By using a vesselness map to provide tubular structure information as prior knowledge to the deep learning model, they recorded improved performance (DSC: 0.8) over existing CNN-based methods. This multi-channel approach demonstrates that filtered images can serve as a crucial guide for deep learning models to learn vascular features within complex backgrounds.

However, the segmentation of the STAs and FVs remains an underexplored area in the field of automated medical image analysis. These vessels entail unique computational challenges: their cross-sectional areas are extremely small relative to voxel resolution, their 3D trajectories are complex, and they are adjacent to high-intensity skeletal structures. These factors lead to severe partial volume effects and intensity heterogeneity, which pose significant obstacles for standard segmentation algorithms in accurately identifying these vessels.

Combining the structural enhancement properties of vesselness filters with the superior feature extraction capabilities of deep learning—as validated in previous studies—is a highly effective strategy for microvascular segmentation [15,16,17]. To automatically segment the small and tortuous STAs and FVs from CTA volumes, this study proposes an approach that generates a fusion-based vesselness map (FVM) with enhanced vascular structures and incorporates this into the loss function design of a deep learning model.

The primary contributions of this study are summarized as follows:

1. We propose an FVM-guided training strategy that incorporates a vesselness-based auxiliary loss into nnU-Net v2, specifically designed to emphasize fine vascular features and improve the accurate delineation of the STAs and FVs.
2. We demonstrate the feasibility of automatically segmenting highly variable and complex superficial vessels, extending the conventional deep learning focus from major central arteries to specific small-caliber structures required in specialized surgeries.
3. We establish a practical foundation for advanced intraoperative guidance, where the proposed automated extraction pipeline offers the potential to enhance surgical precision and contribute to reducing times in overall clinical workflow.

To validate the proposed method, we detail the generation of the FVM and its integration into the nnU-Net v2 architecture. The segmentation performance is then evaluated on clinical CTA datasets by comparing the FVM-guided loss against standard loss functions and other baseline models. Based on the quantitative and qualitative results, we analyze the clinical feasibility of the proposed framework and discuss its potential to improve visualization in complex neurosurgical, facial, and reconstructive surgeries.

## 2. Materials and Methods

**Figure 1:**
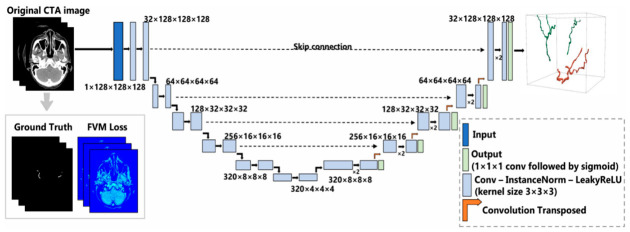
Overview of the proposed vessel segmentation framework.

In this section, we describe the proposed framework for the automatic 3D segmentation of STAs and FVs in CTA images. The overall workflow of our method is illustrated in Figure 1. To achieve robust segmentation, our pipeline processes the input CTA volumes by applying boundary-aware filtering to generate the FVM for enhanced vascular contrast, followed by automated 3D deep learning segmentation. Detailed descriptions of each module are provided in the following subsections.

### 2.1. Vesselness Map Generation

To effectively enhance vascular structures, the FVM was constructed by integrating multiple structural features. In this framework, the standard Frangi filter [18] was initially applied to highlight tubular structures; however, because it relies on the eigenvalue analysis of the Hessian matrix, it exhibits reduced responses at bifurcations and within small-vessel regions [19]. To overcome this limitation, we propose a novel Vesselness Score derived from the linear fusion of multiple filters to reinforce fine and branching vascular structures.

Building upon this improved score, the comprehensive FVM was developed by integrating multiple vessel enhancement features categorized into three distinct groups: edge masks, vesselness filters, and exclusive masks. The edge masks included a gradient magnitude map and adaptive thresholding to emphasize vascular boundaries. The vesselness filters comprised the proposed vesselness scoring components and structure-response filtering to reinforce tubular continuity. Additionally, the exclusive mask applied HU-based constraints and TotalSegmentator-derived bone and skin-boundary masks to suppress non-vascular responses from air, bony structures, and surface artifacts [20]. Each map was independently generated based on distinct imaging characteristics and subsequently combined through a weighted fusion process to produce the final integrated FVM.

For each voxel k the Vesselness Score was defined as:

(1) $${Vesselness\;Score}_{k}=\;\omega_{GMM}\cdot S_{k}^{GMM}+\omega_{VF}\cdot S_{k}^{VF}+\omega_{AT}\cdot S_{k}^{AT}$$

where $S_{k}^{GMM}$, $S_{k}^{VF}$, and $S_{k}^{AT}$ denote the signal intensities from the gradient magnitude map, the vesselness filter, and the adaptive thresholding map, respectively [16,19,21,22]. The weights $\omega_{GMM}$, $\omega_{VF}$, and $\omega_{AT}$ could be adjusted to appropriate values depending on the dataset characteristics. In particular, the vesselness filter could yield either positive or negative responses depending on the characteristics of the filter signal. In this formulation, the weight is set to a positive value when the filter exhibits high precision and to a negative value when it demonstrates high recall.

To improve the response at vascular bifurcations and small-vessel regions where the standard Hessian-based Frangi filter [18] often yields reduced outputs [19], the filtering components were further modulated. Specifically, the multiscale Frangi filter was configured with vessel radius candidates of 0.25, 0.5, 1.0, 1.5, and 2.0 mm to fully span the expected diameters of target vessels, such as the STAs and FVs, in craniofacial CTA. Furthermore, a vesselness bias of +0.1 was applied to regions with a vesselness response above 0.5 to promote tubular structures. Conversely, a penalty of −0.2 was assigned to regions outside the adaptive edge mask to strongly suppress background noise.

To eliminate anatomically irrelevant regions and false-positive responses, a spatial exclusive mask, $M_{k}∊\;\{0,1\}$, was applied. Let Ω be the entire voxel set. The mask $M_{k}$ was set to 0 if voxel k was located within non-vascular regions defined by HU-based constraints or TotalSegmentator-derived anatomical masks. Specifically, air-like regions were excluded using an HU threshold of (k < −25) HU, whereas bony structures were excluded using a bone mask generated by TotalSegmentator. Surface-related false-positive responses were further suppressed by applying a 2.0 mm skin-boundary constraint layer derived from the TotalSegmentator skin mask. Otherwise, ($M_{k}$ = 1). The final FVM was obtained by normalizing the masked Vesselness Score as follows:

(2) $${FVM}_{k}=\;M_{k}\cdot \frac{{Vesselness\;Score}_{k}}{{max}_{k\epsilon \Omega }\left({Vesselness\;Score}_{k}\right)}\;$$

### 2.2. Dataset

This retrospective study was approved by the Ethics Committee of Asan Medical Center (Approval number: 2026-0018). Patient data were fully anonymized, and the requirement for informed consent was waived by the committee, in compliance with relevant ethical guidelines. CTA datasets were acquired from patients who underwent reconstructive plastic surgery procedures in the Department of Plastic Surgery at Asan Medical Center. All CTA scans were performed using a SOMATOM Definition Edge scanner (Siemens Healthineers, Erlangen, Germany) at 80 kV and 309 mA.

Among 75 CTA cases initially collected, three cases were excluded due to severe dental metal artifacts that precluded reliable identification of the facial vessel course. The remaining 72 cases were divided into a development pool of 52 cases and an independent test set of 20 cases. Within the development pool, a five-fold cross-validation scheme was implemented, where 10 cases were randomly selected for validation in each fold, and the remaining cases were allocated for training. The test set of 20 cases was excluded from the training and hyper-parameter tuning phases, serving solely as an independent cohort for final evaluation.

CTA volumes with varying slice thicknesses were resampled via interpolation to a uniform slice thickness of 0.5 mm prior to analysis. Ground-truth segmentation masks were created in 3D Slicer [23] by two biomedical imaging experts, each with more than five years of experience. To establish a single consensus ground truth, the 72 CTA cases were equally divided between the two experts for initial 3D segmentation. Following this initial phase, a cross-review process was implemented, wherein each expert reviewed the annotations produced by the other. Any disagreements were iteratively refined and adjudicated through joint discussion until both experts mutually approved the final masks for all cases. Throughout the annotation and review process, both experts were blinded to the patients’ clinical records and surgical outcomes.

### 2.3. Network Architecture

The segmentation model in this study was built upon the nnU-Net v2 framework proposed by Isensee et al. [14], and the 3D full-resolution configuration was adopted in this work. The network backbone consisted of a six-stage 3D U-Net with channel widths of (32, 64, 128, 256, 320, 320) across successive stages. All convolutional layers employed 3 × 3 × 3 kernels, and the encoder used strides of (1, 2, 2, 2, 2, 2) for progressive downsampling. Each stage comprised two convolutional layers followed by instance normalization and LeakyReLU activation. The decoder was symmetrically connected to the encoder via skip connections.

To better capture the fine vascular structures of the STAs and FVs, FVM was incorporated into the training procedure. FVM was generated from each input CT image to enhance vascular centerline visibility and was integrated into the loss function to guide the model toward accurate segmentation of thin and tortuous vessels (Figure 1).

### 2.4. Objective Function

Dice Focal Loss was adopted as the baseline owing to its effectiveness in handling severe class imbalance, a common challenge in vessel segmentation in which background voxels vastly outnumber foreground voxels. Both configurations were trained from scratch under identical settings to ensure a fair comparison. To place additional emphasis on thin and tortuous vascular structures, the proposed FVM loss was designed by incorporating vesselness-weighted supervision. The overall objective function ($L_{Total}$) is formulated as follows:

(3) $$L_{Total}\;=\;L_{DiceFocal}\left(P,\;Y_{GT}\right)\;+\;\lambda \;L_{FVM}\left(P,\;FVM_{k}\right)$$

where λ represents the weighting parameter for the auxiliary vesselness loss, which was set to 0.5. P denotes the predicted probability map of the network, $Y_{GT}$ represents the discrete ground-truth labels, and $FVM_{k}$ represents the continuous vesselness map.

Both $L_{DiceFocal}$ and $L_{FVM}$ utilize an identical core loss formulation, $L_{basis}\left(P,\;Y\right)$, which combines Soft Dice loss ($L_{Dice}$) and Soft Focal loss ($L_{Focal}$):

(4) $$L_{basis}\left(P,\;Y\right)\;=L_{Dice}\left(P,\;Y\right)\;+\;L_{Focal}\left(P,\;Y\right)$$

The constituent loss terms are defined as follows. The Soft Dice loss is formulated as:

(5) $$L_{Dice}\left(P,\;Y\right)\;=\;\frac{2\;\Sigma_{k\in \Omega }\;\left(p_{k}y_{k}\right)}{\Sigma_{k\in \Omega }\;\left(p_{k}\right)\;+\;\Sigma_{k\in \Omega }\left(y_{k}\right)}$$

The Soft Focal loss handles both binary and continuous targets by applying an interpolated modulating weight ($\omega_{k}$) to the standard binary cross-entropy ($BCE_{k}$) loss:

(6) $$L_{Focal}\left(P,\;Y\right)\;=\;\frac{1}{N}\sum_{k\in \Omega }\left(\omega_{k}\cdot BCE_{k}\right)$$

(7) $$BCE_{k}=-\left[y_{k}log\left(p_{k}\right)+(1-y_{k})log\left(1-p_{k}\right)\right]$$

where the focal weight $\omega_{k}$ is computed by interpolating the target probabilities

(8) $$p_{target,\;k}=p_{i}y_{i}+(1-p_{k})(1-y_{k})$$

(9) $$\alpha_{target,\;k}=\alpha \;y_{i}+\left(1-\alpha \right)\left(1-y_{k}\right)$$

(10) $$\omega_{k}=\alpha_{target,\;k}{\left(1-p_{target,\;k}\right)}^{\gamma }$$

Here, $p_{k}\in P$ and $y_{k}\in Y$ denote the predicted probability and the target value at voxel k, respectively. N is the total number of voxels within the volume. The hyper-parameters for the focal term, γ and α, were set to 2.0 and 0.25, respectively.

While $L_{DiceFocal}$ evaluates the network output against discrete binary targets ($y_{k}\in \left\{0,1\right\}$), $L_{FVM}$ directly utilizes the continuous voxel-wise values from $FVM_{k}$($y_{k}\in \left[0,1\right]$) as soft targets. For binary targets, this focal formulation mathematically reduces to the original discrete Focal Loss. For continuous soft targets, it seamlessly provides spatially interpolated weighting to enforce structural and spatial continuity along the vascular trajectories.

### 2.5. Experiments

Experiments were conducted on a Linux workstation equipped with an AMD Ryzen 7 7800X3D (4.20 GHz) CPU, NVIDIA GeForce RTX 4090 GPU, and 64 GB RAM. The subsequent analyses and implementations were performed in Python 3.11.

To identify the optimal segmentation model, four architectures were compared: 3D U-Net, Swin UNETR, U-Mamba, and nnU-Net v2. All models were trained under identical conditions and evaluated on the validation set using DSC, IoU (Intersection over Union), Precision, and Recall [15,24,25]. The model with the best overall performance was selected for subsequent experiments.

Using the selected model architecture, two loss functions were compared: the Dice Focal Loss and the proposed FVM loss (detailed in Section 2.4). Both configurations were trained from scratch under identical settings to ensure a fair comparison. To validate its efficacy, model performance was evaluated on the test set using standard metrics, including the DSC and Average Symmetric Surface Distance (ASSD) [15]. To rigorously assess the structural and topological integrity of the segmented vessels, we employed specific measures across three structural dimensions. First, centerline alignment was quantified using overlap (OV) [26] and the 95th percentile Hausdorff Distance (HD95) [27] to assess how closely the predicted and ground-truth vessel skeletons align. Second, branch detection performance was evaluated using precision, recall, and F1-score to measure how accurately the model identifies vascular bifurcations and branching points [28,29]. Third, topological consistency was measured via the centerline Dice (clDice) to quantify the centerline overlap between the predicted and ground-truth vessels, and via the 0th Betti number error [30] to calculate the difference in the number of connected components, reflecting vessel disconnections. Additionally, Grad-CAM was utilized as a qualitative explainable AI method to evaluate model interpretability [31].

### 2.6. Qualitative Clinical Evaluation

To evaluate the clinical applicability of the segmentation models, a blind visual assessment was conducted by two clinical evaluators. A 5-point Likert scale (1: completely unacceptable to 5: excellent) was used to score the 3D segmentation results of 20 cases. The evaluation was based on three criteria: (1) morphology (overall 3D trajectory and anatomical correctness); (2) noise/FP suppression (absence of false positives like skull, skin, or non-target vessels); and (3) surgical planning efficacy (readiness for use in 3D visualization and surgical planning). The predicted volumes from both the baseline and the proposed method were presented in a randomized and blinded manner.

## 3. Results

### 3.1. Model Selection

**Figure 2:**
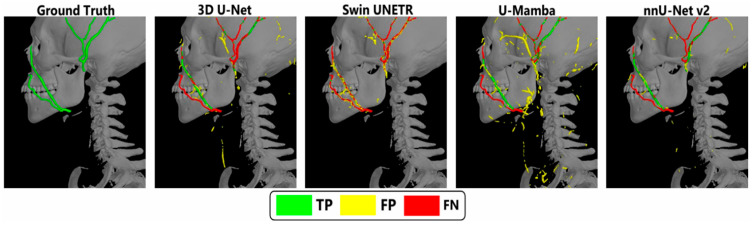
Qualitative comparison of vessel segmentation performance among four architectures. Green, yellow, and red denote true positives (TP), false positives (FP), and false negatives (FN), respectively.

To identify the most appropriate base model for vessel segmentation, four architectures were compared: 3D U-Net, Swin UNETR, U-Mamba, and nnU-Net v2. Among them, nnU-Net v2 achieved the best quantitative performance across all evaluation metrics, with a DSC of 0.624, IoU of 0.458, Precision of 0.625, and Recall of 0.639 (Table 1). In the qualitative analysis, 3D U-Net and U-Mamba tended to generate noisy predictions, whereas Swin UNETR produced relatively cleaner outputs but showed more false-positive regions than nnU-Net v2 (Figure 2). Considering both the quantitative and qualitative results, nnU-Net v2 was selected as the base model for subsequent experiments.

### 3.2. Effect of FVM Loss on Segmentation Performance

#### 3.2.1. Quantitative Evaluation

Table 2 summarizes the quantitative results. When using only the Dice Focal Loss, the DSC values for the STAs and FVs were 0.621 and 0.634, and the ASSD values were 6.543 mm and 4.238 mm, respectively. With the addition of the FVM loss, the DSC values for the STAs and FVs were 0.643 and 0.637, and the ASSD values were respectively 2.941 mm and 2.130 mm, indicating an overall improvement in boundary precision. The relatively low DSC of the FVs was attributed to the model’s tendency to respond more sensitively to venous structures while being less accurate in segmenting arterial regions. Nevertheless, the model incorporating the FVM Loss demonstrated improved continuity and morphological consistency along vessel boundaries compared with the Dice Focal Loss baseline.

#### 3.2.2. Qualitative Evaluation

**Figure 3:**
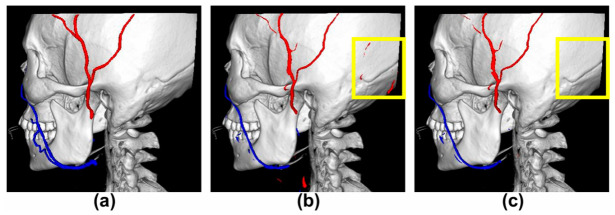
Comparison of the STAs and FVs segmentation results in CTA using different loss functions. (**a**) Ground truth; (**b**) Dice Focal Loss; (**c**) FVM Loss. Red indicates the STAs, and blue indicates the FVs.

For qualitative evaluation, the ground truth masks and predicted segmentation masks were reconstructed in 3D and visualized together with the CT bone model (Figure 3). When using only the Dice Focal Loss, the model was able to capture the overall spatial distribution and coarse morphology of the STAs and FVs with reasonable agreement with the ground truth. However, the predictions contained noticeable segmentation noise, including spurious detections and boundary irregularities, particularly in distal and branching regions. These errors were frequently observed in low-intensity vascular areas or regions with intensities similar to those of surrounding tissues, indicating limited discrimination in such challenging regions.

In contrast, the model trained with the additional FVM Loss produced smoother, cleaner predictions with reduced false positives. Predictions adhered more closely to anatomical vessel boundaries and showed a selective suppression of non-target superficial vessels adjacent to skin and skull (e.g., occipital scalp vessels), while preserving the intended STAs/FVs targets.

**Figure 4:**
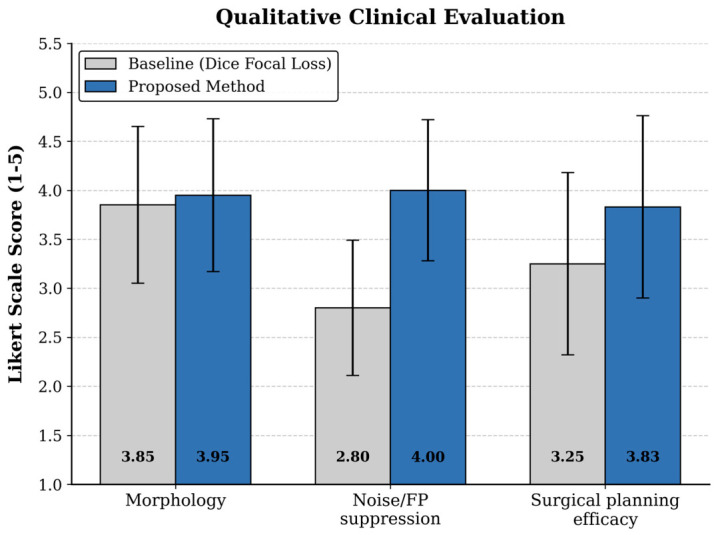
Qualitative clinical evaluation results based on a 5–point Likert scale.

These visual improvements were quantitatively supported by the clinical evaluation results (Figure 4). The proposed method consistently outperformed the baseline across all clinical criteria. Most notably, our model demonstrated an improvement in noise/FP suppression, increasing the average score from the baseline’s 2.80 to 4.00. This confirms our visual findings that the proposed method mitigates the over-segmentation of non-target structures. Consequently, this noise reduction translated into enhanced surgical planning efficacy (3.25 vs. 3.83), indicating that our segmentation results require less manual post-processing and are more readily applicable for preoperative surgical planning. Furthermore, the morphological accuracy was well-preserved and showed improvement (3.85 vs. 3.95).

**Figure 5:**
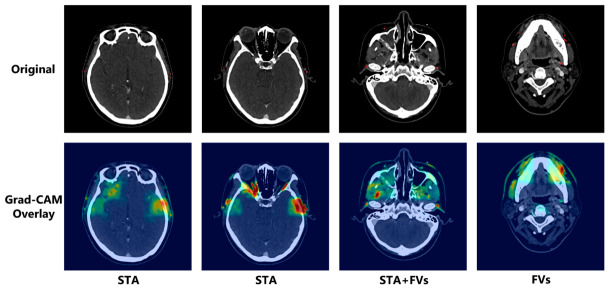
Grad-CAM visualization of the segmentation network under the proposed loss function. (**Top row**) Original craniofacial CTA slices and (**bottom row**) corresponding Grad-CAM activation overlay maps.

To qualitatively evaluate the explainability of the model, Grad-CAM was applied to visualize the regions that primarily influenced predictions (Figure 5). In successfully predicted cases, the regions exhibiting high activation precisely aligned with the fine anatomical boundaries of the bilateral STAs. This continuous and localized activation pattern demonstrates the efficacy of the proposed vesselness map loss, indicating that the decoder successfully learned the target vascular topology.

Conversely, a localization bias was observed in some cases, where the Grad-CAM heatmaps were asymmetrically activated near adjacent mandibular boundaries and the cranial base, rather than being evenly distributed across the bilateral vascular branches. This phenomenon indicates that although the proposed FVM loss was designed to capture continuous linear features down to small-caliber vascular segments, its heightened sensitivity also responded to non-vascular structures with similar geometric scales, such as the optic nerve bundle or thin soft-tissue interfaces.

Consequently, during backpropagation, high positive gradient weights were assigned to these regions. This localization bias represents a technical trade-off resulting from the aggressive optimization toward fine vessel continuity, where non-target anatomical pathways with vessel-like geometric properties were inherently highlighted.

#### 3.2.3. Ablation Study

To determine the optimal loss weight λ for the auxiliary vesselness loss, comparative experiments were conducted across various λ values (Table 3). The experimental results demonstrated that the model achieved the best overall performance at λ = 0.5, resulting in a DSC of 0.640, precision of 0.716, and ASSD of 2.53 mm. Although the highest recall of 0.652 was observed at λ = 0.1, λ = 0.5 was selected for the final model to ensure an optimal balance across all performance metrics.

Furthermore, to quantitatively evaluate the contribution of each component within the FVM generation framework, an ablation study was conducted across four configurations (Table 4). The baseline model, which utilized only the conventional vesselness filter (Case 1), achieved a DSC of 0.634 and an HD95 of 19.588 mm. Incorporating the exclusive masks (Case 2) substantially improved the branch detection rate, yielding the highest recall (0.749) and F1-score (0.653) while reducing the boundary error to an HD95 of 15.650 mm. Conversely, integrating the edge masks without the exclusive masks (Case 3) resulted in an increased DSC of 0.644, though it exhibited the lowest precision (0.561) in branch detection.

Our proposed configuration, which integrates all three components, demonstrated a slightly lower DSC (0.640) than Case 3 but achieved the highest precision (0.632) in branch detection. Furthermore, in terms of centerline distance, it achieved the highest overlap (OV) of 0.828 while still maintaining a low HD95 of 10.171 mm. In terms of topology-aware metrics, our model surpassed the other configurations by achieving the highest clDice (0.799) and the lowest 0th Betti number (20). These results indicate that the comprehensive FVM framework efficiently suppresses false-positive components while preserving the connectivity of complex, small-caliber vessels. Consequently, the combination of all components was selected as the optimal FVM configuration for subsequent experiments.

## 4. Discussion

In this study, a deep learning framework was developed to automatically segment the STAs and FVs from craniofacial CTA. These vessels are thin, tortuous, and adjacent to high-contrast bony structures, resulting in a significant intensity overlap and partial volume effects that hinder clear differentiation. To determine the optimal architecture, 3D U-Net, Swin UNETR, U-Mamba, and nnU-Net v2 were evaluated. While 3D U-Net and U-Mamba produced noisy predictions and Swin UNETR yielded high false positives, nnU-Net v2 demonstrated the most stable quantitative and qualitative performance. Consequently, nnU-Net v2 was selected as the baseline model.

To improve the segmentation of the STAs and FVs, which are small-caliber branches, an FVM was incorporated into the training loss function of the nnU-Net v2 model. The FVM combines classical image processing operators, including multiscale vesselness filters. Compared to the baseline model trained solely with the Dice Focal Loss, the FVM-guided model achieved superior surface consistency, as evidenced by the improved ASSD. Specifically, the proposed method reduced the ASSD by 55.05% (from 6.543 mm to 2.941 mm) for the STAs and by 49.74% (from 4.238 mm to 2.130 mm) for the FVs, demonstrating a substantial improvement in boundary precision. Within the loss function, the FVM acts as a spatially varying weight. The FVM emphasizes tubular shapes and the distinct borders between vessels and adjacent tissues, while suppressing non-vascular regions like the inside of bones and the skin surface.

This weighting strategy mitigates the over-confident predictions [32,33,34] typically associated with Dice or focal-based objectives. Qualitatively, the FVM-guided loss also suppressed non-target superficial vessels in the occipital region. Because the FVM relies on tubularness and boundary-based responses, non-target vessels with low signal-to-noise ratios and extremely small calibers fail to generate strong responses and are subsequently suppressed during training. This indicates a detectability bias toward more conspicuous vascular segments, which proved beneficial for isolating the target STAs and FVs in this study.

Several limitations exist in this study. First, the framework was evaluated on a relatively small, single-center dataset consisting of 72 clinical cases. Although five-fold cross-validation and an independent test set of 20 cases were utilized, the single-institution design restricts the generalizability of the findings across different CTA scanners, slice thicknesses, and imaging protocols. Second, volume- and surface-based metrics such as DSC and ASSD do not fully capture the connectivity of small terminal branches. A model might achieve a high DSC while missing surgically critical distal segments. Third, the precomputed FVM does not explicitly differentiate between arteries and veins. Because it remains fixed during training, its ability to adapt to diverse imaging conditions or anatomical variations is restricted.

Future work will focus on expanding the generalizability and task-specific performance of this framework. External validation using multicenter datasets is planned, along with the application of the pipeline to other thin arterial structures, such as hepatic, renal, and internal iliac branches. Centerline- and node-based evaluation metrics will also be incorporated to better assess branch preservation and topology [35,36]. Finally, integrating the automated segmentation results with 3D-printed models, surgical navigation, and augmented reality platforms [37,38] will allow for the evaluation of the practical impact of this approach on operative time and surgical precision in clinical settings.

## 5. Conclusions

An automated deep learning framework was developed for the precise segmentation of the STAs and FVs in craniofacial CTA by integrating an FVM into the nnU-Net v2 loss function. Compared to the baseline model trained solely with the conventional Dice Focal Loss, the proposed FVM-guided approach significantly improved boundary precision and substantially reduced symmetric surface distance errors for both target vessels. This spatial weighting strategy effectively emphasized continuous tubular structures, suppressing non-target superficial vessels and segmentation noise in distal branches. By reliably isolating these challenging small-caliber vessels, the proposed method successfully addresses the limitations of manual annotation and establishes a practical foundation for advanced surgical planning, navigation, and augmented reality integration in clinical settings.

## Figures and Tables

**Table 1:** Comparison of vessel segmentation performance among different deep learning models.

Model	DSC	IoU	Precision	Recall
3D U-Net	0.485	0.323	0.516	0.473
Swin UNETR	0.534	0.365	0.531	0.546
U-Mamba	0.406	0.258	0.326	0.574
nnU-Net v2	0.624	0.458	0.625	0.639

**Table 2:** Quantitative evaluation of segmentation performance for the STAs and FVs using DSC and ASSD.

Loss	Vessel	DSC	ASSD (mm)
Dice Focal	STA	0.621	6.543
	FVs	0.634	4.238
Dice Focal + FVM (ours)	STA	0.643	2.941
	FVs	0.637	2.130

**Table 3:** Performance comparison across different loss weights (λ). Data are presented as mean (standard deviation).

Weight	DSC	Precision	Recall	ASSD
0.1	0.638 (0.092)	0.674 (0.119)	0.652 (0.153)	3.274 (2.726)
0.3	0.628 (0.089)	0.714 (0.102)	0.602 (0.150)	2.722 (1.535)
0.5 (ours)	0.640 (0.092)	0.716 (0.107)	0.621 (0.154)	2.535 (1.292)
0.7	0.639 (0.096)	0.709 (0.116)	0.624 (0.157)	2.726 (1.833)
0.9	0.637 (0.093)	0.705 (0.106)	0.624 (0.155)	3.014 (2.074)
3.0	0.640 (0.095)	0.707 (0.110)	0.628 (0.157)	2.817 (1.800)

**Table 4:** Ablation study of FVM components on vessel enhancement performance. Data are presented as mean (standard deviation).

Method	Components			Label Average	Branch Detection Rate			Centerline Distance		Topology- Aware	
	Vessel -Ness Filter	Edge Masks	Exclusive Masks	DSC	Precision	Recall	F1	HD95 (mm)	OV	clDice	0th Betti
case1	O	-	-	0.634 (0.082)	0.583 (0.221)	0.682 (0.221)	0.607 (0.192)	19.588 (24.643)	0.816 (0.092)	0.787 (0.093)	29 (14)
case2	O	-	O	0.631 (0.087)	0.605 (0.248)	0.749 (0.206)	0.653 (0.218)	15.650 (16.823)	0.817 (0.093)	0.787 (0.094)	30 (15)
case3	O	O	-	0.644 (0.093)	0.561 (0.230)	0.678 (0.227)	0.582 (0.186)	10.129 (9.653)	0.826 (0.091)	0.797 (0.096)	27 (14)
ours	O	O	O	0.640 (0.092)	0.632 (0.252)	0.694 (0.249)	0.625 (0.202)	10.171 (8.035)	0.828 (0.087)	0.799 (0.092)	20 (11)

## Data Availability

The datasets generated and/or analyzed during the current study are not publicly available due to institutional restrictions and patient privacy regulations but are available from the corresponding author upon reasonable request and with permission from the Institutional Review Board of Asan Medical Center.

## Abbreviations

The following abbreviations are used in this manuscript:

STA	Superficial temporal artery
FVs	Facial vessels
CTA	Computed tomography angiography
FVM	Fusion-based Vesselness Map
GT	Ground Truth
ASSD	Average symmetric surface distance
DSC	Dice similarity coefficient
